# Quantitative analysis of axon collaterals of single pyramidal cells of the anterior piriform cortex of the guinea pig

**DOI:** 10.1186/s12868-017-0342-7

**Published:** 2017-02-08

**Authors:** Junli Yang, Gerhard Litscher, Zhongren Sun, Qiang Tang, Kiyoshi Kishi, Satoko Oda, Masaaki Takayanagi, Zemin Sheng, Yang Liu, Wenhai Guo, Ting Zhang, Lu Wang, Ingrid Gaischek, Daniela Litscher, Irmgard Th. Lippe, Masaru Kuroda

**Affiliations:** 1 Second Affiliated Hospital of Heilongjiang University of Chinese Medicine, Harbin, 150001 China; 2 Department of Anatomy, School of Medicine, Toho University, Tokyo, 143-8540 Japan; 3 Research Unit for Complementary and Integrative Laser Medicine, Research Unit of Biomedical Engineering in Anesthesia and Intensive Care Medicine, and TCM Research Center Graz, Medical University of Graz, 8036 Graz, Austria; 4 Privatclinic Lassnitzhoehe, 8301 Lassnitzhoehe, Austria; 5 Institute of Experimental and Clinical Pharmacology, Medical University of Graz, 8036 Graz, Austria

**Keywords:** Anterior piriform cortex, Olfactory cortex, Olfactory bulb, Single neurons, Axon collaterals, Neural network

## Abstract

**Background:**

The role of the piriform cortex (PC) in olfactory information processing remains largely unknown. The anterior part of the piriform cortex (APC) has been the focus of cortical-level studies of olfactory coding, and associative processes have attracted considerable attention as an important part in odor discrimination and olfactory information processing. Associational connections of pyramidal cells in the guinea pig APC were studied by direct visualization of axons stained and quantitatively analyzed by intracellular biocytin injection in vivo.

**Results:**

The observations illustrated that axon collaterals of the individual cells were widely and spatially distributed within the PC, and sometimes also showed a long associational projection to the olfactory bulb (OB). The data showed that long associational axons were both rostrally and caudally directed throughout the PC, and the intrinsic associational fibers of pyramidal cells in the APC are omnidirectional connections in the PC. Within the PC, associational axons typically followed rather linear trajectories and irregular bouton distributions. Quantitative data of the axon collaterals of two pyramidal cells in the APC showed that the average length of axonal collaterals was 101 mm, out of which 79 mm (78% of total length) were distributed in the PC. The average number of boutons was 8926 and 7101, respectively, with 79% of the total number of boutons being distributed in the PC. The percentage of the total area of the APC and the posterior piriform cortex occupied by the average distribution region of the axon collaterals of two superficial pyramidal (SP) cells was about 18 and 5%, respectively.

**Conclusion:**

Our results demonstrate that omnidirectional connection of pyramidal cells in the APC provides a substrate for recurrent processes. These findings indicate that the axon collaterals of SP cells in the PC could make synaptic contacts with all granule cells in the OB. This study provides the morphological evidence for understanding the mechanisms of information processing and associative memory in the APC.

**Electronic supplementary material:**

The online version of this article (doi:10.1186/s12868-017-0342-7) contains supplementary material, which is available to authorized users.

## Background

The piriform cortex (PC) has long been treated as the “primary” olfactory cortex because of the largest area that receives direct input from the olfactory bulb (OB) [1, 2], the structure that monosynaptically relays input from olfactory neurons [3]. It is not homogeneous in structure although the entire PC has the same basic three-layer organization.

Many differences in both axonal connections and the cytoarchitecture of different regions of the PC have been described [1, 4–10]. It is generally considered to consist of just two divisions though Rose [11] subdivided the rodent PC into multiple areas. The PC is divided into the anterior piriform cortex (APC) and the posterior piriform cortex (PPC) [6, 12, 13]. The most obvious difference between the APC and the PPC is that the lateral olfactory tract (lo) stops short of the PPC. APC and PPC also differ in the organization of intrinsic associational systems. Although cellular-level analysis will be required for confirmation, population-level morphological studies indicate that associational axons are both rostrally and caudally directed in the APC, and largely caudally directed in the PPC [3, 6, 9, 12, 13]. Physiological and modeling analysis has shown that the afferent activation of the APC is fast compared to the duration of postsynaptic potentials [14, 15]. A study from 2001 reported that the APC can be divided into dorsal (APC_D_) and ventral (APC_V_) subdivisions [10]. The PPC is situated posterior to the LOT and recognizable by a well-developed layer III, and the APC_D_ is located dorsal to the LOT with a cytoarchitecture that is somewhat intermediate between that of the APCv and that of the PPC. These differences in structure are believed to reflect differences in functional roles [10, 16]. The structure of the APC has led to the hypothesis that the PC functions as a distributed processing neural network and is critically involved in information processing and associative memory [17–19].

The current view of the odor discrimination suggests that the APC serves as a site of experience-induced enhancement in odorant discrimination, indicating convergence of odor information from many kinds of odor receptors into one PC neuron [20–29]. Studies by Wilson demonstrated that APC neurons discriminate alkane odorants based on carbon chain length [22, 23]. A mapping study of c-fos immunoreactivity in response to odorants suggested odor-specific spatial patterns of activity within the APC [30]. A result from optical imaging studies suggests that the dorsal part of the APC may be associated with odor concentration [31]. Therefore, in addition to recruitment of more olfactory sensory cells and glomeruli in response to stronger stimuli, a rostro-caudal gradient in axonal projections from mitral/tufted cells and/or in association fibers may play an important role in odor-concentration coding in the APC.

Studies using extracellularly-injected axon tracers have shown that associational axons are widely distributed spatially in the PC and extend into many adjacent cortical areas. However, these connections are not distributed at random; rather, there are broad, overlapping spatial patterns in both the origins and terminations of association axons [6, 9, 12, 13, 32]. An intracellular labeling study of pyramidal cells in layer IIb of the rat PPC showed cellular-level connectivity [3]. A surprise from this analysis is that individual layer II pyramidal cells in the PPC have extensively branching axons that are distributed to most of the highest-order behavior-related areas in the cerebral cortex. Previous studies also revealed the axonal branching patterns and bouton distribution of individual neurons in layers IIa, IIb, and III in the guinea pig PC [33–35]. These studies explored the organization of olfactory information processing in the APC, and investigated the validity of the current view of odor discrimination in the APC. In the present study using an intracellular injected axonal tracer, we will quantitatively analyze the distribution of axon collaterals on individual neurons in the APC, with special reference to the following points: (1) Analysis of the number of PC neurons which make synaptic contacts with single APC neurons. Through this analysis, we can estimate the number of different kinds of odor receptors, which converge information into one APC neuron. (2) Analysis of the number of synapses of one APC neuron in the OB. By this analysis, we can estimate the magnitude of synaptic contacts between axon collaterals of APC neurons and granule cells of the OB. This study examines features of axon connections of superficial pyramidal (SP) cells that provide the morphological evidence for understanding the mechanisms of information processing and associative memory in the APC.

## Methods

### Experimental procedures

Animal procedures were approved by the Toho University Animal Care and Use Committee and conformed to the animal use guidelines of the National Institute of Health. Thirty-one male and female adult guinea pigs (800–1000 g/per animal) were anesthetized with intraperitoneal urethane injection (1 g urethane/kg body weight). Briefly, each animal was mounted on a stereotaxic instrument (Narishige, SN-3). Drainage of the cerebrospinal fluid at the atlantooccipital joint was routinely carried out to minimize pulsation of the brain. An opening in the dorsal cranium was made using a dental drill to introduce stimulating electrodes to the OB. Another opening was made for recording electrodes in the middle and the anterior part of the PC. The exposed surfaces of the brains were covered with a mixture of warmed mineral oil and white vaseline to prevent cooling and drying of the brain. Body temperature was maintained at approximately 37 °C using a heating pad. Stimulating bipolar concentric electrodes were vertically inserted into the anterior part of the OB. A glass recording electrode filled with 0.5 M KCI and 4% biocytin (Sigma, St. Louis, MO, USA) in 0.05 M Tris buffer, pH 7.4, was vertical inserted into the PC from the dorsal surface of the neocortex. To identify the position of the microelectrode tips in the PC, we monitored the field potential evoked by OB stimuli, whose A_1_-peak wave reversed near the border between layers I and IIb [20, 36–38]. Cells were impaled in an area between the reversal point of the field potential and a point 100 μm deeper than the reversal point. The injection of biocytin was performed by passing 1-3 nA depolarizing pulses for 500 ms at a frequency of 1.0 c.p.s., for 10–35 min. After biocytin injection, the wound was sterilized and sutured (Additional file 1: Table 1).

### Histology

6-12 h After the injection of biocytin, animals were perfused through the heart with 4% paraformaldehyde in 0.1 M sodium phosphate buffer (PB), pH 7.4. The brain was excised, postfixed overnight, and cryoprotected in 20% sucrose in PB. Serial frozen sections were cut at 80 µm thickness. Sections were incubated with avidin–biotin-peroxidase complex (ABC; Vector, Burlingame, CA, USA). Biocytin-labeled cells were stained by incubation with 0.035% 3-3′-diaminobenzidine (DAB, Sigma) solution, and 0.015% H_2_O_2_ in 0.5 M Tris buffer, pH 7.4. The sections were mounted on gelatin-coated slides, and counterstained with 0.05% thionin.

### Data analysis

Analysis was confined to SP cells in the dorsal subdivision of the APC [12, 13, 39]. The soma and dendrites were traced and reconstructed in coronal planes using a Nomarski-type microscope (Olympus) equipped with a drawing tube using a 40 × objective. Axons were reconstructed through serial coronal sections using 20, 40, and 100 × objectives. The reconstructions were rotated and superimposed onto the illustrations of the brain surface. The lengths of the axonal segments of each area in each coronal section were measured using a pen-type map meter (Koizumi; accuracy of measurement, ± 1 mm).

For the quantitative determination of axon length, shrinkage in depth was corrected on the basis of the original section thickness of 80 μm. However, shrinkage in other dimensions was not corrected because it was minimal due to the attachment of sections to slides before dehydration. Accordingly, final axonal length was estimated from the measured length and the 80 μm section thickness. Interbouton intervals were measured in each area at 40× magnification. Boutons were identified using the criteria established in an electron microscopic analysis of intracellularly injected pyramidal cells in the opossum PC [40].

Brain areas were defined according to previous descriptions [36, 37, 41–43]. The boundaries of the PC layers were defined according to the description of Price [1] and Haberly and Price [2, 32].

Statistical significance was analyzed by the student *t* test and Welch’s *t* test when populations differed. Photomicrographs were acquired digitally using an Olympus SZX 12 microscope fitted with an Olympus DP 50 camera using Viewfinder Lite software (version 1.0). Image quality was optimized by adjusting sharpness using Paintshop Pro 9 software (version 7.04: Jasc Software) (Additional file 2: Table 2).

## Results

To explore the organization of olfactory information processing and investigate the validity of view of the odor discrimination in the APC by using an intracellular biocytin, we injected axonal tracer into SP cells in the APC of the guinea pig.

**Fig. 1:**
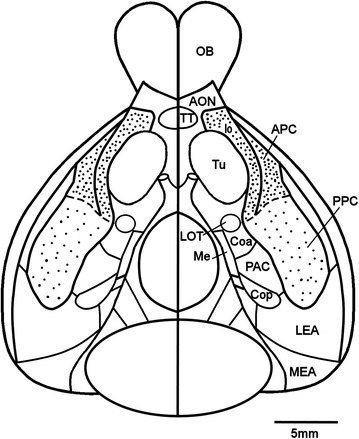
Reconstruction of the olfactory areas on a ventral view of the guinea pig brain. The boundary between anterior piriform cortex (*APC* heavy stippling) and posterior piriform cortex (*PPC* light stippling) is indicated by the *dashed line*

The PC is commonly separated into APC and PPC, as illustrated in Fig. 1.

SP cells have a pyramidal to ellipsoid soma. These cells exhibit a single apical dendrite whose branches extend to the superficial limit of the molecular layer, multiple basal dendrites, a high concentration of dendritic spines, and a deeply directed axon [6].

**Fig. 2:**
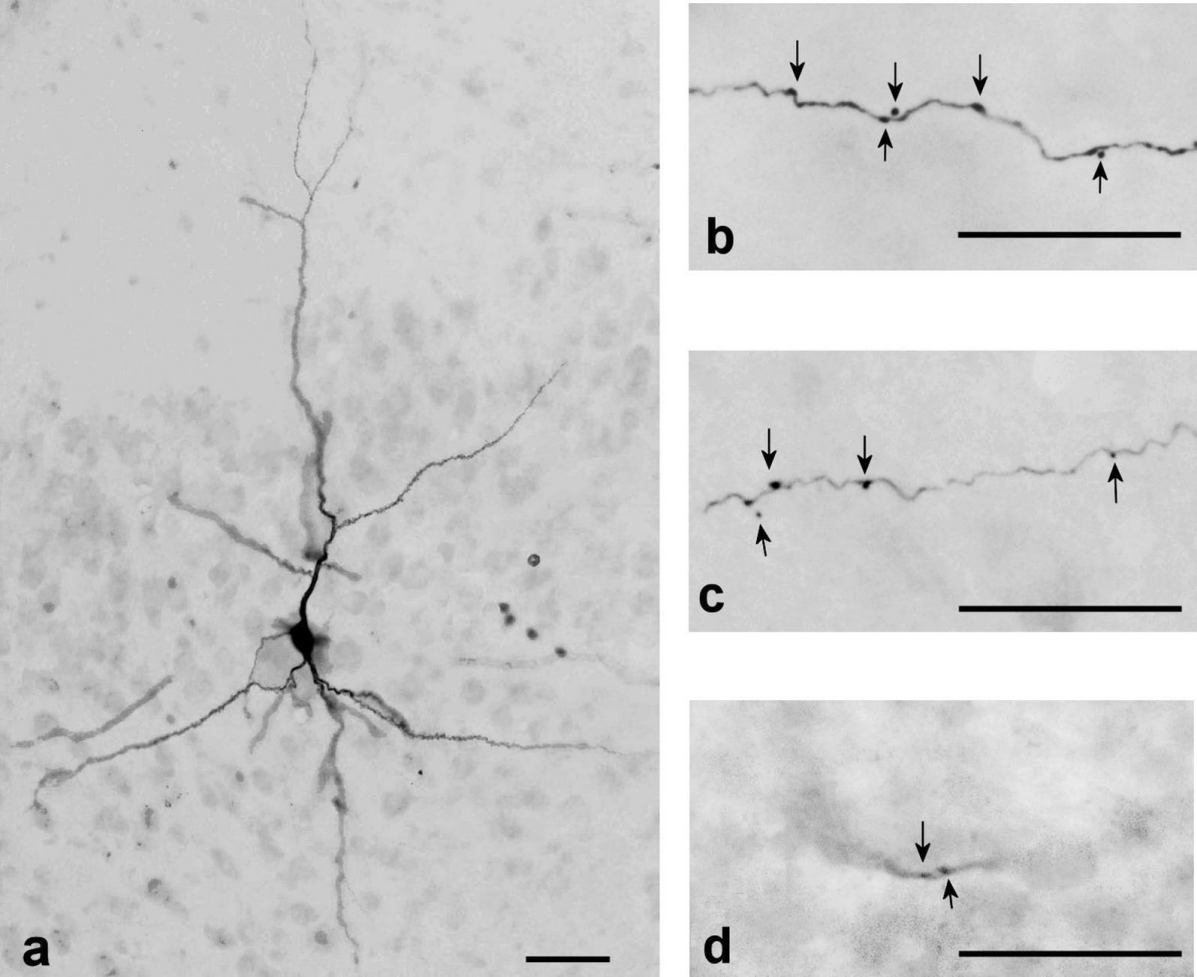
Photomicrographs of coronal sections of the piriform cortex of the guinea pig. **a** Biocytin-injected superficial pyramidal cell in layer IIb. **b**–**d** Axon collateral of superficial pyramidal cell in layer III, AON and OB, respectively. *Arrowheads* indicate boutons. *Scale bar* = 50 μm

In this study, the total number of stained SP cells in the APC was fifteen; reconstructions and detailed analysis were performed on two of the cells that appeared to be representative of the population because of difficulties in serially reconstructing the extensively arborized axon collaterals that were 90-111 mm long. Because of difficulties in the serial reconstruction of the extensively arborized axon collaterals, two SP cells, located in the caudal portion of the APC, were reconstructed and quantitatively analyzed. Examples of a biocytin-labeled SP cell and its axon collaterals are shown in Fig. 2.

### Distribution of axon collaterals

**Fig. 3:**
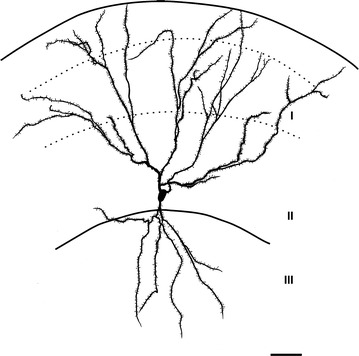
Camera lucida illustration of soma and dendrites of a superficial pyramidal cell in the APC (SP-1 cell). Dendrites were reconstructed in a coronal plane. *Solid lines* indicate the surface of the brain and border between layers IIb and III, and *dashed lines* indicate the border between layers Ia and Ib and the border between layers Ib and IIa. Note the large number of small spines on both the apical and basal dendrites. *Scale bar* = 50 μm

The SP cells had a pyramidal to ellipsoid soma, of which the long and short axes were 18.7 ± 2.1 and 12.1 ± 0.8 µm (mean ± SEM, n = 2). They exhibited a single apical dendrite the branches of which reached layer Ia and multiple basal dendrites. Both apical and basal dendrites were covered with a large number of small spines as shown in Fig. 3. The mean length of the dendritic spines of the two SP cells was 1.26 ± 0.07 µm (mean ± SEM, n = 104). The axon originated from the soma and projected 7–8 major collaterals. The lengths of axons from the soma to the end of branching points of major collaterals ranged from 255 to 335 µm (280 ± 55 µm, n = 2).

**Fig. 4:**
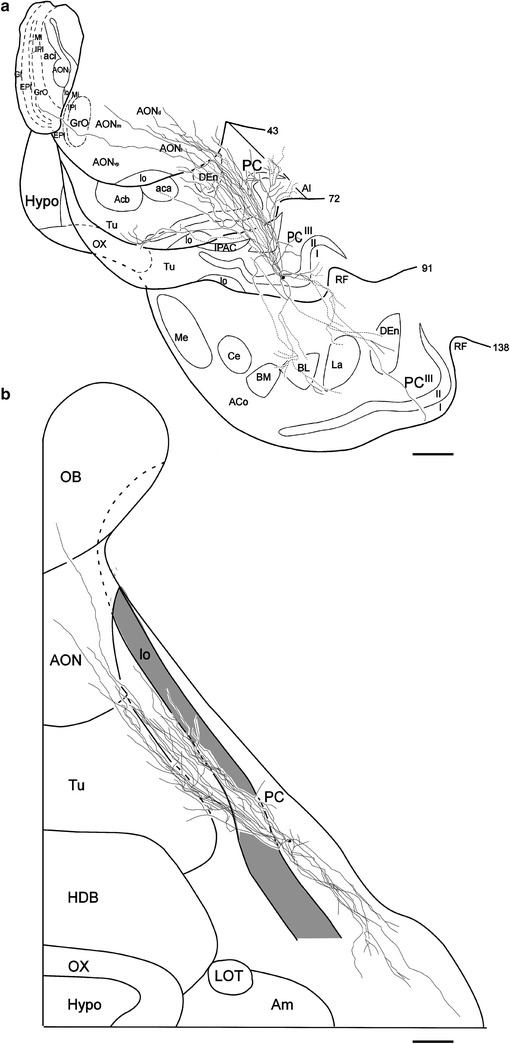
Coronal (**a)** and surface (**b)** views of reconstructed axon collaterals of a superficial pyramidal cell (SP-1 cell) located in the left hemisphere APC. a The position of the soma is indicated by a *small dot*, and that of the axon collaterals by *thin solid* or *dashed lines*. The Arabic numerals indicate the number of sections; the Roman numerals indicate the layer of the piriform cortex (PC). The *solid lines* indicate the axon collaterals distributed in the indicated areas within the represented sections as well as the adjacent sections. The *dashed lines* indicate collaterals running between these sections. Note that the axon collaterals are widely distributed within the PC and other olfactory areas and a long axon collateral projects to the granular cell layer of the olfactory bulb. **b** The small dot indicates the position of the soma. The collaterals (*thin solid lines*) extend to both rostral and caudal directions. The *thick solid lines* indicate the border between the areas of the cortical surface. The *shaded area* shows the lo. *Scale bar* = 1 mm

The SP-1 cell shown in Fig. 4 was located in the APC, 2300 µm rostral to the end of the lateral olfactory tract (lo). The axon collaterals of the SP-1 cell were reconstructed in coronal (Fig. 4a) and tangential planes (Fig. 4b). The axon projected 7 major collaterals to layer III. The axon from the soma to the end of the branching points of the major collaterals was 255 µm long. The major axon collaterals followed relatively straight paths in layer III and gave rise to a small number of short branches and many extent arbors at irregular intervals. Then, some of the axon collaterals were oriented toward the superficial part of the PC, passing through layer IIb, and giving rise to horizontal branches in layer Ib, a small number of which reached layer Ia. The arbors distributed within the PC were directed both rostrally and caudally, but were predominantly rostrally directed, as shown in Fig. 4b. The arbors in the PC were distributed over a wide area up to its medial border and laterally extended up to the rhinal fissure (RF). Outside the PC, the axon collaterals were distributed in the dorsal endopiriform nucleus (DEn), olfactory tubercle (Tu), agranular insular cortex (AI) and anterior olfactory nucleus (AON). In addition, one fairly thick collateral emanating from a major collateral in the deep part of layer III, reached rostrally in layer III adjacent to the DEn. Then, this thick collateral passed through the dorsal part of the AON, and reached the granular cell layer (GrL) of the OB.

**Fig. 5:**
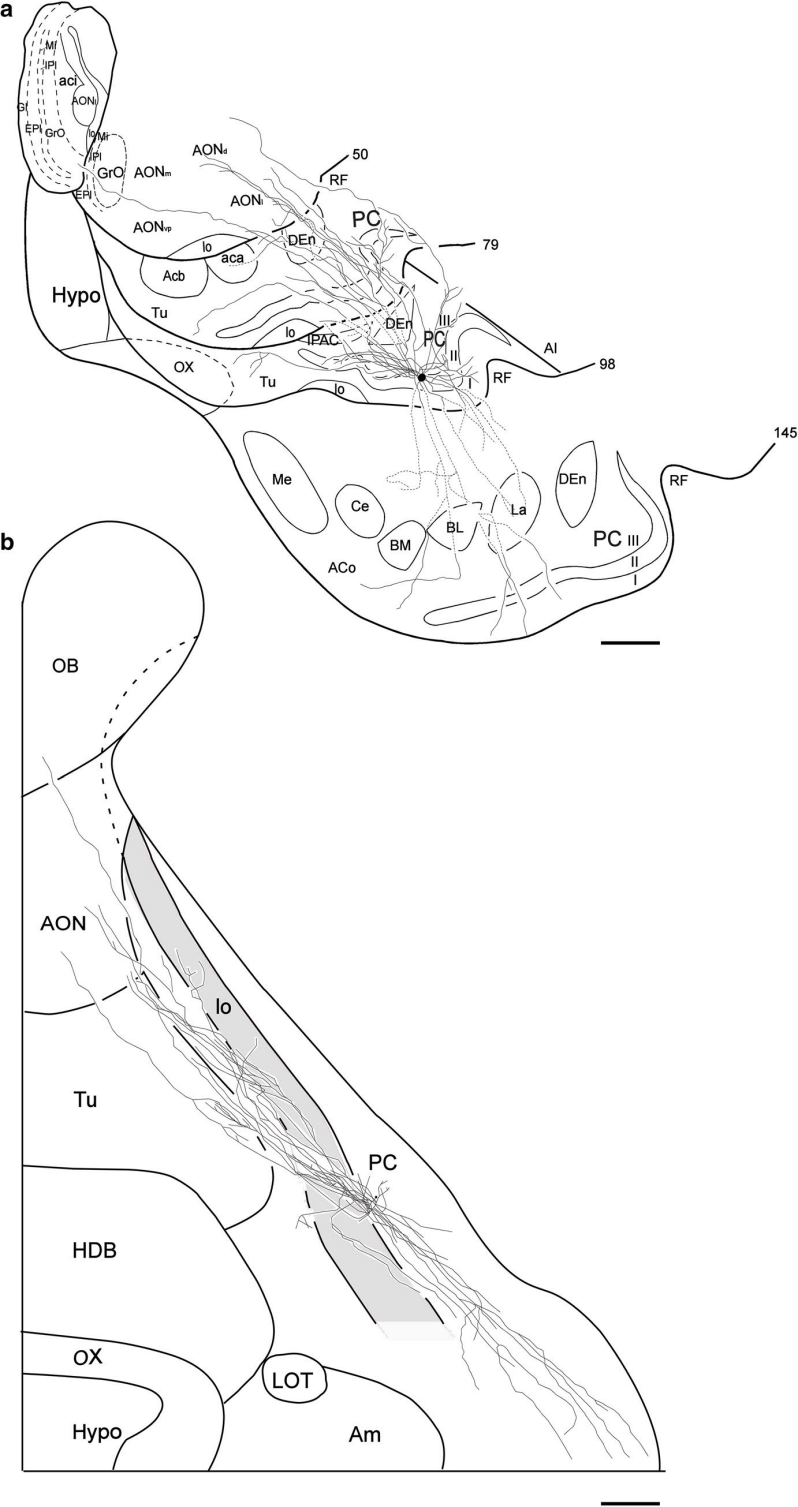
Coronal (**a**) and surface (**b**) views of reconstructed axon collaterals of a superficial pyramidal cell (SP-2 cell) located in the left hemisphere APC. Compared with the collaterals of the SP-1 cell (Fig. 4), the collaterals of this cell are very similar. **a** The axon collaterals are widely distributed within the PC and other olfactory areas and a long axon collateral projects to the olfactory bulb. **b** The *small dot* indicates the position of the soma. The collaterals extend in both rostral and caudal directions. The *thick solid lines* indicate the border between the areas of the cortical surface. *Scale bar* = 1 mm

The SP-2 cell, shown in Fig. 5, was located in the APC, 2100 µm rostral to the anterior end of lo. The axon collaterals of the SP-2 cell were reconstructed in coronal (Fig. 5a) and tangential planes (Fig. 5b). The axon projected 8 major collaterals to layer III. The axon from the soma to the end of the branching points of the major collaterals was 335 µm long. The axon collaterals exhibited routes and distribution patterns largely similar to those of the SP-1 cell. In the tangential plane, the axons distributed within the PC were also directed rostro-caudally, but predominantly rostrally directed, as shown in Fig. 5b. Their collaterals were heavily distributed in the PC. Outside the PC, collaterals also projected to the DEn, Tu, AI and AON. In addition, one fairly thick collateral emanating from a major collateral, took a similar course towards the GrL of the OB.

Table 1 shows the summary of quantitative data on the length of axon collaterals of reconstructed SP cells in the APC. The total length of axon collaterals of the two cells studied ranged from approximately 90–111 mm, with an average length of 101 mm. Axon collaterals with a length of 79 mm (78%) were distributed in the PC and those with a length of 21 mm (22%) in the areas outside the PC. Within the PC, 46-mm-long (57.8%) axon collaterals were distributed in layer III, 9-mm-long axon collaterals (12%) in layer II, 5-mm-long axon collaterals (7%) in layer Ia, and 18-mm-long axon collaterals (23.2%) in layer Ib.

**Table 1:** Length (μm) of axons of neurons in the layer IIb of the APC

Area	Length of axon				Mean length of axon (mean ± SEM)	
	cell-1		cell-2			
Pir Ia	6864	(6.2)	4125	(4.6)	5494 ± 1370	(5.5)
Pir Ib	23,959	(22)	12,624	(14)	18,292 ± 5667	(18.2)
Pir II	10,306	(9.3)	8540	(9.5)	9423 ± 883	(9.4)
Pir III	47,713	(43)	43,365	(48)	45,539 ± 2174	(45.3)
PC total	88,842	(80)	68,654	(76)	78,748 ± 32,641	(78.3)
DEn	4003	(3.6)	6244	(6.9)	5123 ± 1121	(5.1)
Tu	7207	(6.5)	6897	(7.6)	7052 ± 155	(7.0)
AI	3847	(3.5)	2283	(2.5)	3065 ± 782	(3.1)
AON	6147	(5.6)	6052	(6.7)	6099 ± 48	(6.1)
OB	684	(0.6)	174	(0.2)	429 ± 55	(0.4)
Total	110,730	(100)	90,303	(100)	100,517 ± 10,213	(100)

Values in parentheses indicate percentage of length of axons distributed in each area

Outside the PC, 5 mm (5.1%) of the total axon length was distributed in the DEn, 7 mm (7%) in the Tu, 3 mm (3.1%) in the AI, 6 mm (6.1%) in the AON, and 0.4 mm (0.4%) in the OB.

### Distribution of boutons

Table 2 shows the summary of the interbouton intervals and the number of boutons distributed in each area. Axon collaterals provided many boutons. More than 90% of the boutons were boutons en passant, and terminal boutons accounted for a small percentage in each area. The two SP cells did not differ significantly with regard to interbouton intervals in each layer of the PC. The mean interbouton intervals in each layer of the PC were not significantly different from each other. The mean interbouton interval in all layers of the PC was 10.7 ± 0.2 μm (n = 212). The interbouton intervals were 9.4 ± 0.6 μm (mean ± SEM, n = 14) in layer Ia, 10.7 ± 1.2 μm (n = 67) in layer Ib, 11.1 ± 1 μm (n = 44) in layer II, and 11.5 ± 1.3 μm (n = 88) in layer III, which were not significantly different from each other. Outside of the PC, the interbouton intervals in the DEn (13.1 ± 0.6 μm, n = 28), Tu (10.3 ± 0.7 μm, n = 21), AI (12.1 ± 0.8 μm, n = 18) and AON (14.2 ± 1.2 μm, n = 43) were not significantly different from those in the PC. The interbouton interval in the OB (6.6 ± 0.7 μm, n = 7) was significantly shorter (p < 0.05) than that in the PC.

**Table 2:** Number of boutons in neurons in layer IIb of the APC

Area	Interbouton intervals (mean ± SEM)	No. of boutons				No. of boutons (mean ± SEM)	Percentage of boutons en passant
		cell-1		cell-2			
PC Ia	9.4 ± 0.6	730	(7.4)	439	(5.5)	585 ± 145.5	91.3
PC Ib	10.7 ± 1.2	2237	(22.6)	1180	(14.8)	1708 ± 528.6	89.5
PC II	11.1 ± 1	928	(9.4)	769	(9.7)	849 ± 79.5	93.9
PC III	11.5 ± 1.3	4149	(41.9)	3771	(47.5)	3960 ± 189	93.3
PC total	10.7 ± 0.2	8044	(81.2)	6159	(77.9)	7101 ± 942.6	94.4
DEn	13.1 ± 0.6	306	(3.1)	477	(6)	391 ± 85.3	95.9
Tu	10.3 ± 0.7	700	(7.1)	670	(8.4)	685 ± 15.2	94.4
AI	12.1 ± 0.8	318	(3.2)	189	(2.4)	254 ± 64.5	97.2
AON	14.2 ± 1.2	433	(4.4)	426	(5.4)	430 ± 3.5	96.7
OB	6.6 ± 0.7	104	(1.1)	26	(0.3)	65 ± 39	100
Total		9905	(100)	7946	(100)	8926 ± 979.5	

Values in parentheses indicate percentage of number of boutons distributed in each area

The mean total number of boutons in a single SP cell of the APC was 8926. The number of boutons distributed in the PC was 7101 (79.5% of the total number of boutons), out of which 55.8, 24.1, 11.9 and 8.2% were located in layers III, Ib, II, and Ia, respectively.

Outside the PC, the number of boutons was 1825 (20.5% of the total number), out of which 391 were in the DEn, 685 in the Tu, 254 in the AI, 430 in the AON, and 65 in the OB (Additional file 3: Table 3).

### Estimation of area where axon collaterals of a single SP cell are distributed

**Fig. 6:**
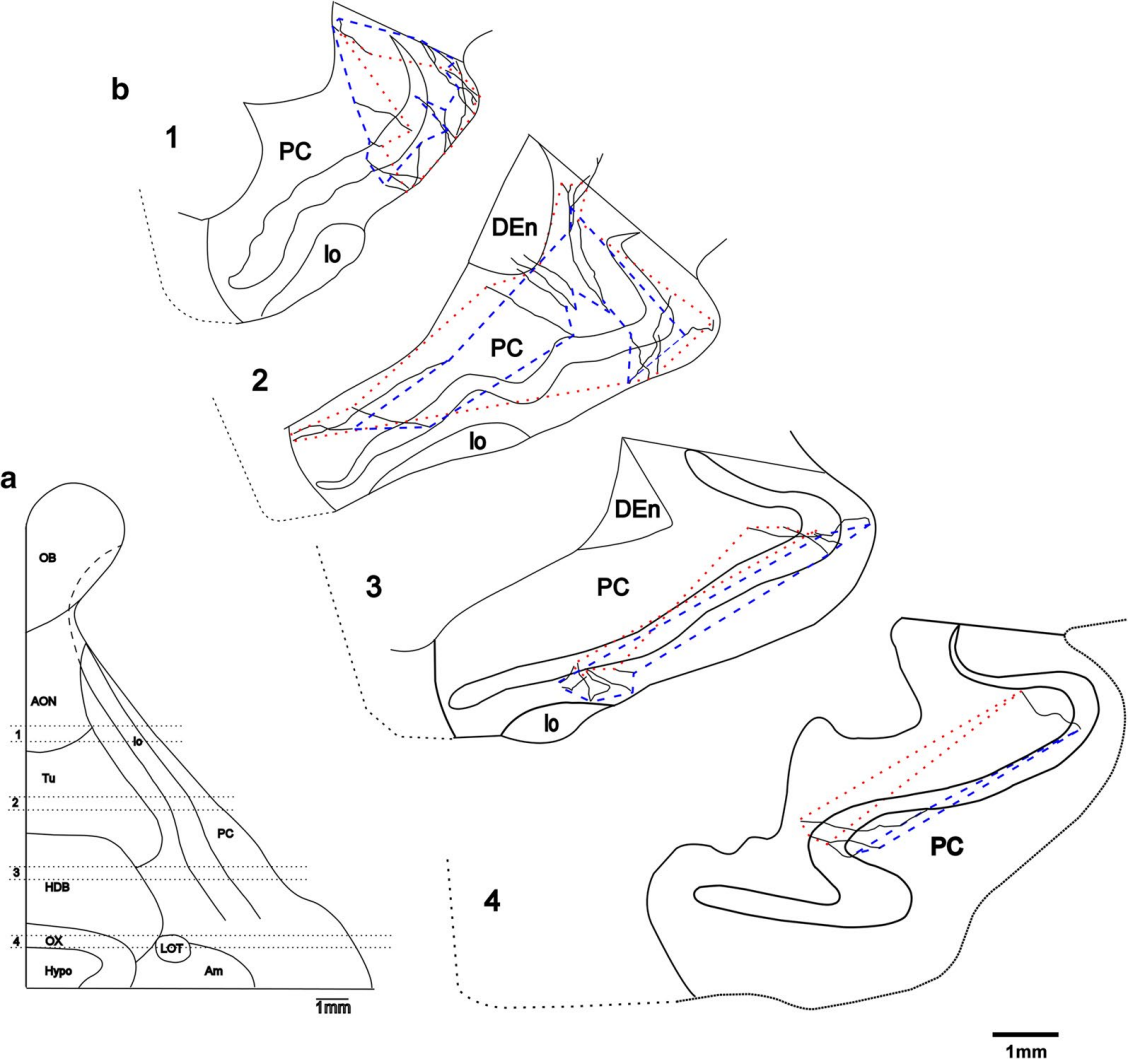
Extent of area where axon collaterals of a single SP cell distribute. Levels 1–4 in the ventral brain surface (**a**) correspond to the four levels in the coronal plane reconstructed from 5 serial sections (**b**). The axon collaterals of the SP-1 cell are distributed within areas interconnecting the outermost tips of proximal ends (*dotted lines*) and those of distal ends (*chain lines*) of the collaterals in each plane

An important question for understanding the nature of information processing is the extent of the area where axon collaterals of single SP cells are distributed. As shown in Figs. 4b, 5b, the axon collaterals of each SP cell followed rather linear trajectories in two conical volumes, of which the apexes were located at the cell body of the SP cell. Assuming that linear trajectories of axon collaterals are randomly distributed in the conical volumes, the area occupied by axon collaterals of one SP cell can be estimated by the areas enclosed by a line interconnecting the outermost tips of each collaterals in the following four levels of the PC: A level of the APC approximately 1800–2200 μm in depth (level 1 in Fig. 6a), a level of the APC approximately 4000–4400 μm in depth (level 2), a level of the APC approximately 6200–6600 μm in depth (level 3), and a level of the PPC approximately 8400–8800 μm (level 4) from the anterior end of the PCs, the total length of which were 9300 μm or 9700 μm in the rostro-caudal direction. In each stack of sections, areas enclosed by a line interconnecting the proximal tips of collaterals and also by a line interconnecting the distal tips were estimated as two areas, where the axon collaterals of single SP cells were distributed (dotted and chain lines in Fig. 6b). The mean distribution areas of the axon collaterals of single SP cells occupied 17.3 ± 4.1% of the APC area in level 1, 28.7 ± 11.7% in level 2, and 8.6 ± 1.0% in level 3, the mean of which was 18.1 ± 4.7% in the APC. The mean distribution area of the collaterals of single SP cells was 4.6 ± 0.9% in level 4 in the PPC (Table 3).

**Table 3:** Percentage of areas occupied by axon collaterals in the piriform cortex

Area	Level	SP-1		SP-2		Mean ± SEM
		Proximal	Distal	Proximal	distal	SP-1 and SP-2
APC	1	20	26	13	10	17.3 ± 4.1
	2	57.8	32.5	13.5	10.6	28.7 ± 11.7
	3	7.4	12.3	4.5	9.9	8.6 ± 1
Mean of APC		28.4	23.6	10.3	10.2	18.1 ± 4.7
PPC	4	5.3	1.3	8	0.5	4.6 ± 0.9

## Discussion

In this study we provided a complete visualization of the associational connections of single pyramidal cells from superficial layers II of APC in the guinea pig by intracellular biocytin injection and quantitative analysis of axon collaterals. The major findings of the present study are: (1) Axon collaterals from single SP cells in the APC are highly branched and widely spatially distributed within the PC and some higher order areas, especially they also showed a long associational projection to the OB. (2) The single SP cell in the APC gives rise to multiple, long association axons, and the long associational axons are both rostrally and caudally directed throughout the PC. Within the PC, associational axons typically followed rather linear trajectories and irregular bouton distributions. (3) The average number of boutons was 8926, with 79% of the total number of boutons (7101) being distributed in the PC, and 0.7% (65) in the OB. (4) The average distribution region of the axon collaterals of the PC of two SP cells occupied about 18 and 5% of the total area of APC and PPC, respectively.

### New features of axon collaterals

The SP cells in the APC in this study have highly spatially distributed axon collaterals in the PC. Previous studies with both extracellularly and intracellularly injected axonal tracers have revealed that the intrinsic associational projections of SP cells in the PC are highly distributed spatially [6, 9, 12, 13, 32, 38, 44–46]. Our results support those of the previous studies. A single SP cell in the APC gives rise to multiple, long association axons, passing throughout the PC. Studies with intracellular injection of SP cells in the PPC in rats [3] showed that axon collaterals are highly branched and distributed over an area that can encompass virtually an entire cerebral hemisphere. This was also shown in our report in the PC of guinea pigs [34]. The present observations showed that axon collaterals from single SP cells in the APC are highly branched and widely spatially distributed. A particularly intriguing feature is that axon collaterals from single SP cells in the APC are more highly distributed in the PC (78% of the total length), and also arborized extensively in some higher order areas within the DEn, Tu and AON. The results are in agreement with those of Johnson et al. [3] and ul Quraish et al. [34] regarding extensive axonal distribution in the PC to the SP cells in the PPC of the rat and to the SP cells in the PC of the guinea pig, but differ in extensive axonal distribution outside the PC. Many studies have shown that long associational axons are both rostrally and caudally directed throughout the APC, and largely caudally directed in the PPC [3, 6, 9, 12, 13]. The studies using an extracellular injected axonal tracer showed the proportion of cells in layers II and III that gave rise to association fibers, and thus explained the predominance of rostrocaudal fibers over caudorostral ones. The results indicate a precise laminar organization of the PC in which the rostrocaudal fibers originate mainly from layer II and the caudorostral fibers primarily from layer III [6, 32]. At the cellular level, our results support the finding that the APC and PPC differ in the organization of the intrinsic association system. The SP cells in the APC have both rostrally and caudally directed intrinsic association fibers. It is proposed that, as a result of this spatially distributed recurrent connectivity, the APC supports autoassociative processes [47]. The results of the present study are also consistent with those of Johnson et al. [3], because within the PC, associational axons typically followed linear trajectories, and regions of extensive branching were observed. Specifically, a long axon collateral projecting to the GrL of the OB was observed in the present study. Data reported on the basis of HRP retrograde transport experiments showed that the APC projects to the OB in the tree shrew [48], cat [49] and rat [6, 32]. The examination of anterograde transport from HRP and amino acid injections into the PC indicate that this projection is to the GrL of the OB [6, 32, 50]. Autoradiographic studies have revealed that neurons of both layer II and layer III in the PC in the hamster have centrifugal projections to the main OB [12, 13, 51–53]. The finding that axon collaterals of single SP cells in the PPC of the rat are projections to the OB has been briefly described by Johnson et al. [3]. Our report confirmed these observations. Furthermore, in this study, this was analyzed quantitatively. Our study is the first to provide a visualization of the association axons projecting to the GrL of the OB from single SP cells of the APC in the guinea pig using intracellular biocytin techniques.

### Information processing in PC

The APC and PPC exhibit many differences in terms of both axonal connections and cytoarchitecture [6, 9, 10, 32]. These differences in structure are believed to reflect differences in functional roles [10, 54]. Despite the fact that the APC is the primary sensory cortex in the olfactory system, relatively little is known about the basic sensory processing of this structure. The structure of the APC has led to the hypothesis that the PC functions as a distributed processing neural network, and is critically involved in information processing and associative memory [17–19]. Considerable attention has been given to the spatial organization of cellular interconnections in the APC. We have demonstrated that individual SP cells in the APC have highly extensive axon collaterals. A particularly intriguing feature is that axon collaterals are widely distributed in the PC, and also arborized extensively in some higher order areas. A striking feature of the APC is its extensive intrinsic associational circuitry that is both rostrally and caudally directed over long distances. It is proposed that, as a result of this spatially distributed recurrent connectivity, the APC supports autoassociative processes [47].

The present study has revealed that the axon collaterals of individual SP cells are distributed in an area occupying approximately 18% of the APC, indicating that information activities from three to four different types of olfactory receptor converge onto individual SP cells in the APC. These anatomical features could facilitate the increase in the synaptic strength of the axon collaterals of SP cells by temporal convergence of co-occurring odor-features, generating the synthetic coding of familiar odors. The synthetic coding of odors as unique objects may increase the discrimination of similar objects as well as enhance recognition of those objects even if input is partially degraded [20–26, 28, 47].

In this study, the axon collaterals of SP cells in the APC projected to the GrL of the OB, although the axon collaterals provided several collaterals reaching the AON. Several workers [6, 12, 13, 32, 50, 51] have reported that the PC projects to the GrL of the OB. The centrifugal projections to the OB originate predominantly in the APC and gradually decrease in number in the PPC. The intrabulbar axon collaterals of single SP cells have been briefly described by Johnson et al. [3]. However, the number of intrabulbar boutons of the collaterals of individual SP cells has not yet been determined. The present study has shown that SP cells in the APC have 26–104 boutons in the GrL of the OB. The number of granule cells in the OB ranges from 2.5 × 10^6^ to 5 × 10^6^ in the rat [54] and from 5 × 10^6^ to 10 × 10^6^ in the rabbit [55]. Assuming that half the population of SP cells in all regions of the PC projects to the GrL of the OB [6, 32, 53], 50% of the intrabulbar boutons of single SP cells form synaptic contacts with granule cells [40] and two boutons located within a short distance (approximately 6.6 µm) form synaptic contacts with one granule cell, the number of boutons required to drive all granule cells is 17–69, which is roughly similar to the number of intrabulbar boutons determined in this study. The intrabulbar axon collaterals of the SP cells elicit monosynaptic EPSP in the granule cells, which in turn inhibits the activity of the mitral cells in the OB [56–58]. The centrifugal fibers from the SP cells alter the form of oscillatory activity in the OB [59], the spatial amplitude patterns of which were suggested to be odor-specific [60].

Our study has of course also some limitations. We used only two neurons for reconstruction and analysis that appeared representative of the population. Addition of that data would bolster the applicability of the conclusions from the two fully reconstructed cells therefore we will gradually complete this work in the further studies.

## Conclusion

The main results of our study show that omnidirectional connection of pyramidal cells in the anterior part of the piriform cortex (APC) provides a substrate for recurrent processes. It indicates that the axon collaterals of superficial pyramidal (SP) cells in the piriform cortex (PC) could make synaptic contacts with all granule cells in the olfactory bulb (OB). This study provides the morphological evidence for understanding the mechanisms of information processing and associative memory in the APC.

## Additional files

**Additional file 1.** Raw data for Table 1.

**Additional file 2.** Raw data for Table 2.

**Additional file 3.** Raw data for Table 3.

## Abbreviations

aca: anterior commissure, anterior
aci: anterior comm, intrabulbar part
Acb: accumbens nucleus
AI: agranular insular cortex
Am: amygdaloid nuclear complex
AON: anterior olfactory nucleus
AON_l_: anterior olfactory nucleus, pars lateralis
AON_d_: anterior olfactory nucleus, pars dorsalis
AON_m_: anterior olfactory nucleus, pars medialis
AON_vp_: anterior olfactory nucleus, pars ventroposterior
APC: anterior part of piriform cortex
APC_D_: dorsal part of anterior piriform cortex
APC_V_: ventral part of anterior piriform cortex
BL: basolateral amygdaloid nucleus
BM: basomedial amygdaloid nucleus
Ce: central amygdaloid nucleus
DEn: dorsal endopiriform nucleus
EPl: external plexiform layer olfactory bulb
HDB: nucleus of horizontal limb diagonal band
Hypo: hypothalamus
IPAC: interstitial nucleus of posterior limb of anterior commissure
IPl: internal plexiform layer olfactory bulb
lo: lateral olfactory tract
Gl: glomerular layer of olfactory bulb
GrL: granular cell layer
GrO: granular cell layer of olfactory bulb
La: lateral amygdaloid nucleus
LOT: nucleus of lateral olfactory tract
Me: medial amygdaloid nucleus
OB: olfactory bulb
OX: optic chiasm
PC: piriform cortex
PPC: posterior part of piriform cortex
RF: rhinal fissure
SP: superficial pyramidal
Tu: olfactory tubercle
